# Surgical technique used in the UK for native tissue anterior pelvic organ prolapse repair (VaST)

**DOI:** 10.1007/s00192-019-04103-8

**Published:** 2019-09-13

**Authors:** Emily Fairclough, Julia Segar, Jenny Myers, Anthony Smith, Fiona Reid

**Affiliations:** 1 Saint Mary’s Hospital, Manchester Academic Health Science Centre, Manchester University NHS Foundation Trust, Hathersage Road, Manchester, M13 9WL UK; 2 Institute of Human Development, Faculty of Medical & Human Sciences, Manchester Academic Health Science Centre, University of Manchester, Oxford Road, Manchester, M13 9PL UK; 3 Institute of Population Health, University of Manchester, Oxford Road, Manchester, M13 9PL UK; 4 Warrell Unit, St Mary’s Hospital, Manchester University NHS Foundation Trust, Hathersage Road, Manchester, M13 9WL UK

**Keywords:** Native tissue repair, Pelvic organ prolapse, Qualitative research, Surgical technique

## Abstract

**Introduction:**

The PROSPECT study found that outcomes for native tissue and mesh prolapse repairs are similar but mesh repairs have a 10% risk of exposure. The current UK surgical mesh pause has led to renewed interest in native tissue surgery. Previous studies of native tissue anterior repair surgical techniques have been limited by the questionnaire study design. The objective of this study was to describe and categorise native tissue anterior repair surgical techniques.

**Methods:**

This prospective qualitative study used a purposive sampling strategy to recruit surgeons. Data were collected through video-recorded observations of surgery, audio-recorded interviews with surgeons and field notes. The study took place in urogynaecology theatres in 21 UK centres. Thematic analysis was performed using computer-based software and themes of surgical technique were developed.

**Results:**

Thirty consultant surgeons were recruited. In all steps of the anterior repair procedure, infiltration, dissection, method of fascial repair, type and method of suturing and suture placement, surgical technique varied between surgeons. The filming of surgery followed by immediate validation with the surgeons gave greater insight. Surgeons’ terminology to describe techniques varied and the investigators' opinions of the techniques performed were not always consistent with the surgeons' descriptions. The concept of fascia in histological terms was not uniform amongst surgeons.

**Conclusion:**

VaST has demonstrated significant variation in native tissue anterior repair surgical techniques and inconsistency in the terminology used to describe them. These inconsistencies may prevent future meaningful research of prolapse surgery. The variation in technique could affect surgical outcomes and this should be explored further.

## Introduction

Previous studies suggested that surgery using polypropylene mesh could offer a better anatomical cure of pelvic organ prolapse (POP) [1]. A large UK-based surgical randomised controlled trial (RCT) (PROSPECT, PROlapse Surgery: Pragmatic Evaluation and randomised Controlled Trials) [2] was conducted to compare outcomes of native tissue and mesh-augmented repairs. This study showed that the outcomes for both categories of repair are similar but mesh-augmented repairs have an additional 10% risk of mesh complications.

PROSPECT was a pragmatic RCT in which surgeons used the surgical techniques routinely used in their clinical practice. At the start of the study surgeons completed a questionnaire to document their surgical techniques for both native tissue and mesh/graft repairs [3]. This demonstrated significant variation in the surgical technique used to perform an anterior repair. The limitations of the questionnaire study design, including the uncertainty about surgeon’s terminology, gave cause for further evaluation. The current mesh pause adds additional importance to this study.

This prospective qualitative study, Variation in Surgical Technique (VaST), was proposed to gain greater insight into the surgical technique variations that exist and to understand why practice variation continues to exist despite the known importance of evidence-based medicine. The objective of this study was to describe and categorise surgical techniques used to perform native tissue anterior repairs so that future studies can assess the impact of surgical technique on the outcome of surgery.

## Methods

This multi-centered UK-based prospective observational study used qualitative methodology to evaluate the surgical techniques used for native tissue anterior POP repairs. A purposive sample was drawn from a cohort of surgeons who had recruited to the large surgical prolapse study, PROSPECT [2]. This sample was chosen to allow a future subgroup analysis of the influence of surgical technique on outcome. An additional sample of surgeons who had chosen not to participate in PROSPECT was included to ensure techniques were representative of common practice. Recruitment concluded following saturation of themes.

Data collection was performed at the individual surgeons’ hospital site and the surgery was observed during routine theatre schedules. The same investigator performed all interviews and observations. Each surgeon was filmed performing a native tissue anterior repair. This was followed by a face-to-face semi-structured audio-recorded interview with the participating surgeon about their surgical technique. Additional field notes were taken.

All interviews were professionally transcribed in a verbatim manner and a subset sent to the surgeons to ensure accuracy. Thematic analysis [4] using all data was performed and the six phases of analysis were followed. Stages 1–3 (familiarisation with data, generating initial codes and searching for themes) involved two of the investigators. A further investigator was involved in stages 4–6 (reviewing themes, defining and naming themes and producing a report) and in independently reviewing a subset of videos. The computer software (NVIVO) was used in the analysis of data to code and develop themes.

The first objective of VaST was to directly observe, describe and categorise techniques used to perform a native tissue anterior repair. Ethical approval was gained from the Sunderland Ethics Committee (REC no. 13/NE/0158, 29/05/13).

## Results

Thirty surgeons were recruited to VaST; 2 surgeons were interviewed and 28 surgeons interviewed and filmed performing a native tissue anterior repair. These UK-based consultant surgeons worked in 1 of 21 centres (tertiary and district general hospitals) across England and Scotland. Table 1 summarises the background demographics of the surgeons and the procedures performed, both isolated anterior repairs and those with concomitant procedures.

**Table 1:** Demographics of surgeons and details of concomitant surgery

	Number
Type of surgeon	
PROSPECT	22
Non-PROSPECT	8
Gender of surgeon	
Male	20
Female	10
Type of consultant appointment	
General gynaecologist	1
Gynaecologist with special interest	14
Accredited subspecialist in Urogynaecology	14
Urologist	1
Years since consultant appointment	Mean 12 years (range 3–31)
Procedures observed	32
Anterior repair alone	12
Anterior repair + sacrospinous fixation	4
Anterior repair, posterior repair + sacrospinous fixation	4
Anterior and posterior repair	5
Anterior repair, posterior repair and vaginal hysterectomy	6
Anterior repair, posterior repair and Manchester repair	1

## Current techniques used to perform native tissue anterior repairs

### Infiltration

At the start of the procedure most surgeons used infiltration in the anterior wall (*n* = 27/30). However, there was large variation in the volume used, from 3–80 ml (median 20 ml). The type of infiltration used included: local anaesthetic alone; local anaesthetic with saline; local anaesthetic with adrenaline; local anaesthetic with both adrenaline and saline; and finally adrenaline with saline. None used saline alone. The local anaesthetics used include lidocaine, bupivicaine and levobupivaciane.

Surgeons were asked where they injected the infiltration. Some of the surgeons stated that they placed the infiltration within a specific place in either a superficial or deep plane.

Surgeon I: It’s just underneath the vaginal skin. Obviously it is very difficult when you’re infiltrating to judge whether you are underneath the fascia or not but I try to be superficial so that I get a layer between the fascia and the skin.

Surgeon L: That’s an interesting one and we have been arguing for years as to exactly where you are but I think that I am sub-fascial.

Others were less certain of where the infiltration was placed and one surgeon described letting the infiltration, ‘find the plane itself’ (Surgeon AB). The surgeon’s description of the depth of infiltration did not always match the investigators' observations (Table 2).

**Table 2:** Surgeons' and investigators' views of infiltration placement

Placement of infiltration	Surgeon's view	Investigator's view
None	3	3
Superficial	10	8
Deep	12	5
Uncertainty	5	1
Mixed	0	11

Some surgeons used the presence or absence of blanching of the skin to inform whether the infiltration was in the place they wished it to be. Some took the presence of blanching to signify a superficial placement and others were unsure of what blanching signified.

Surgeon Q: I'm infiltrating it so that…the skin goes white. What layer that is, I have no idea, but essentially what I'm trying to do, without any good evidence, is to make it go whiter.

Surgeon R: I inject local and adrenaline in the operation site underneath the fascial layer, so I don’t want to see skin blanching.

### Incision

Most surgeons used a longitudinal midline incision, performed with either a scalpel or scissors. One routinely used an elliptical incision (Surgeon Z) and one used a diathermy pen (Surgeon J).

When considering the caudal aspect of the incision, all surgeons expressed that they avoided the area overlying the urethra. The terminology to describe this landmark varied including ‘bladder neck’; ‘2 cm’, ‘3 cm’ or ‘4 cm below the urethra’; ‘just below the urethra’; ‘urethro-vaginal sulcus’; ‘where rugosity is lost’ and ‘at the extent of the bulge’.

When considering the cephalad extent of the incision most surgeons stated: the cervix or the vault. Other surgeons stated: 1 cm from the cervix/vault, as far as they could reach or to the extent of the prolapse. One surgeon failed to articulate an anatomical landmark and stated, ‘It is related to experience’ (Surgeon J).

### Dissection

**Fig. 1:**
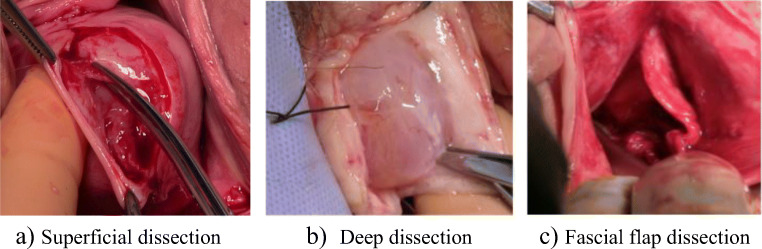
Photographic illustrations of the levels of dissection. **a** Superficial dissection. **b** Deep dissection. **c** Fascial flap dissection

The depth of the dissection through the anterior vaginal wall varied. Some performed a superficial dissection aiming to leave the vaginal muscularis, often called fascia, on the bladder and others described a deep dissection aiming to leave the vaginal muscularis on the vaginal epithelium. Figure 1 shows photographic illustrations of the different depths of dissection. Surgeon F described dissecting the vaginal muscularis from both the vaginal epithelium and the underlying bladder creating ‘fascial flaps’ (Fig. 1c). One surgeon described dissecting to the plane ‘that seems right’ but was unable to specify what this plane was.

The extent of lateral dissection was discussed with the surgeons. Some boney landmarks were described including the pubic arch (Surgeon P1), underneath the pubic rami (Surgeon P2, Surgeons E, F, O, S, AB) and behind the symphysis pubis. Others described muscular landmarks including the white line (arcus tendoneous fascia pelvis, ATFP) (Surgeon A), obturator internus (Surgeons J, Y) and pelvic side wall (Surgeon AA). A proportion of surgeons did not identify a landmark and explained the lateral extent of dissection as being something difficult to articulate or related to a surgeon's intuition.

Surgeon M: I go as far as I think I need to go; that perhaps sounds rather vague and unacceptably vague but that’s what I do.

### Fascial repair methods

Interviews and video analysis identified two levels of fascial repair, which were dependent on the depth of dissection. When a superficial dissection was performed sutures were placed in the vaginal muscularis, which was left attached to the bladder. When deep dissection was performed the sutures were also placed in the vaginal muscularis, which in these cases was left attached to the vaginal epithelium. Superficial fascial repair was the most commonly observed method.

**Fig. 2:**
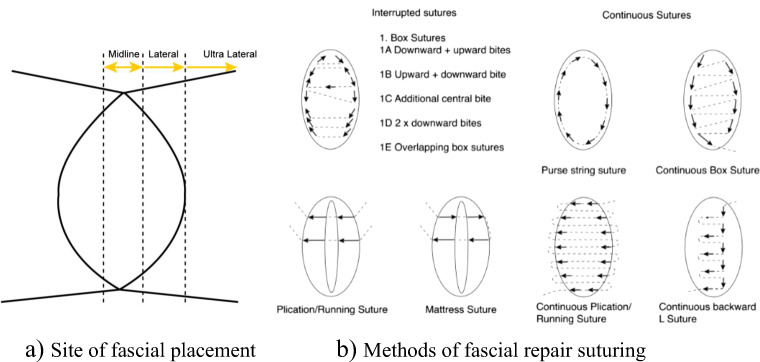
Site of fascial suture placement and methods of fascial repair suturing. **a** Site of fascial placement. **b** Methods of fascial repair suturing

The most common method of repair, no matter the depth or structure repaired, was a form of midline suturing. The number of layers the repair included varied from one to three and the lateral extent of suture placement also varied (Fig. 2). A variety of suturing methods were observed. These methods were either interrupted or continuous and are summarised in Fig. 2. Other methods observed included a superficial dissection followed by repair of isolated defects in the vaginal muscularis repair (separate fascial defects, *n* = 2), placement of sutures in the skin when the vaginal muscularis was left attached to the bladder (n = 2), placement of sutures in the bladder wall when the vaginal muscularis was attached to the vaginal epithelium (n = 2) and repair of fascial flaps.

The suture material used to repair the vaginal muscularis included polygycolic acid suture (PGA) or polydioxanone suture (PDS) and sometimes a combination of both when two or more layers of fascial repair were performed.

### Skin trimming

Skin excision was performed to some extent by nearly all surgeons. Those who did not routinely trim the vaginal skin discussed situations when vaginal skin was excised. Most surgeons who trimmed the vaginal skin stated they were careful only to remove a small amount. Some stated that the amount of skin trimmed was dependent on the amount of redundant skin or the size of the bulge/prolapse. Surgeon AA explained, ‘enough so the skin lies without being under any tension’.

### Skin closure

The vaginal epithelium was closed predominantly with PGA suture but of varying calibres (0, 1, 2.0). One surgeon closed the vaginal epithelium with poliglecaprone 25 (monocryl). The main method of closure was a continuous locking (CL) (*n* = 23/28) suturing. The other methods included continuous non-locking (CNL) (*n* = 2/28), interrupted (*n* = 1/28) and mattress sutures (*n* = 1/28) and one surgeon described a specific suture method called ‘the bunny stitch’ (surgeon S) (*n* = 1/28). It was described as repetitions of three continuous non-locking sutures in the vaginal epithelium followed by a separate interrupted suture that included tissue from the vaginal epithelium and muscularis.

### Surgical terminology

Most surgeons used the term ‘fascia’ to describe the tissue they were repairing. However, the concept of fascia was not uniform amongst surgeons. Some described fascia as being part of the vaginal skin (Surgeon X), others described it as a separate entity (Surgeon L) and some expressed uncertainty about what the tissue was or what planes they were operating in (Surgeon J).

Surgeon X: It’s part of the skin; there are two layers of the fascia.

Surgeon L: Well it’s a layer and you can separate it off the skin.

Surgeon J: I can get into a plane quite comfortably…I think I’m leaving most of the ‘fascia’ on the bladder, but would I describe it as a bladder tissue or a vaginal tissue? I don’t know. Can I sit on the fence on that one?

For the purpose of the above descriptions the histological terms were used to ensure clarity of the plane being described [5]. From observations and later video analysis it was concluded that when surgeons used the term ‘fascia’ this corresponded to the layer of the vagina which in histological terms is called the vaginal muscularis. There were cases however, where the verbal description of infiltration placement and depth of dissection did not match the investigator's view. Despite surgeons' description of infiltration placement in a specific plane, it was the investigators' view that just over a third of surgeons placed the fluid in multiple planes rather than in one distinctive plane (Table 2).

As outlined in the descriptions above, surgeons used varying terms to describe the caudal and cephalic aspects of the incisions, the lateral extent of dissection and the fascial and skin suture methods. The terms used to describe the repair of the vaginal muscularis included plication, buttressing and repair; in some instances these terms were used interchangeably by the same surgeon.

## Discussion

### Main findings

Despite the known importance of evidence-based medicine, the VaST study found significant variations exist between surgeons in the techniques they used to perform anterior repairs. Qualitative methods (video observations and interviews) have allowed categorisation of the entire procedure. The degree of variation seen was greater than had previously been described in the literature when simple questionnaires were used [6–8]. The combination of these variable steps results in potentially hundreds of different types of native tissue anterior repair. VaST is the first study to visually categorise surgical technique hence removing errors related to terminology that are inherent in questionnaire-based studies. It is also the first study to relate the techniques in every step of the procedure with outcome [9].

When performing anterior repairs surgeons did not follow a single method described in the literature [10–12] but instead the techniques used by individuals were a mixture of multiple methods. The categorisation of surgery and development of overarching themes of technique were not possible. The themes developed reflect this and represent the variations seen in the steps of the procedure rather than reflecting the procedure as a whole. The themes of technique include depth of infiltration and dissection, fascial repair method, fascial suture placement, number of fascial repair layers, fascial suture material, fascial suture method, skin trimming, skin suture material and skin suture method.

In a number of cases, there was a difference between the investigator's view of the techniques observed in real time and on video and the techniques described by the surgeons during interview. The lack of agreed terminology to describe these surgical techniques and anatomical landmarks is likely to be a contributing factor. In this group of surgeons the term fascia was commonly used but on further questioning it was poorly defined. In addition there are aspects of technique that surgeons had more difficulty in describing because they were more subjective, the most significant being the extent of lateral dissection. Previous questionnaire studies will not have been able to capture these tacit issues.

### Strengths and Limitations

A key strength of the VaST study is that qualitative methods allowed a greater understanding of the variation of surgical techniques used in an anterior repair procedure. A good sample size was gained (30), at participant 27 saturation of themes was reached and 3 further surgeons were recruited for confirmation. The demographic spread of the surgeons was likely representative of UK practice as a whole.

Video observations have given a perspective that is not possible to gain from questionnaires or interviews alone. The sequence of observation of surgery followed by interview allowed immediate validation of findings with the surgeons and generated areas for discussion. Filming vaginal surgery proved to be relatively easy and it may be useful to include film material in future surgical trials and training.

Surgeons were filmed operating in their own surroundings, hence geographical logistics limited the researcher to one visit per site. This limited the ability to observe variation of technique within the individual surgeon’s practice; however this was discussed in the subsequent interviews with the surgeons. This triangulation of methods should have reduced the impact of this limitation.

### Interpretation

This study has developed themes of surgical technique for native tissue anterior repair. The categorisation of this procedure was not straightforward because of variations existing in all steps of the procedure, inability of surgeons to articulate aspects of their surgical technique and lack of agreement on terminology.

Within the literature there are descriptions of different techniques, which are categorised under the umbrella term of ‘anterior repair’ [6–8, 10–16]. As with previous questionnaire studies [3, 6, 8] this study identified that most surgeons dissect in a superficial plane and this was frequently combined with a midline repair of the vaginal muscularis. This technique was first described by Kelly in 1913 for the treatment of stress incontinence [10]. However most surgeons now avoid the first 3 cm of the anterior vaginal wall, which is a significant variation of technique from that described by Kelly. This is likely due to the indication for anterior repair changing from management of urinary incontinence to a surgery for prolapse.

When reviewing methodology in randomised control trials making an assessment of prolapse repairs, surgical technique variance should be considered a confounding factor on outcome. In the literature, there are reports of a standard anterior repair or midline plication being performed. However, we know from our study that in clinical practice there is considerable variation in each step of the procedure as there is nothing ‘standard’ about repair of the anterior compartment. This highlights the importance of studying surgery in pragmatic trials across multiple centres to ensure the external validity of the results. In the future we would suggest more detailed descriptions of surgical technique.

Surgery consists of explicit and tacit techniques; explicit ones such as a suture type are easy to define and record but tacit techniques such as the extent of lateral dissection are difficult to assess and describe. Video analysis allows us to view aspects of tacit technique not possible from questionnaires or interviews alone. However, the difficulty of teaching tacit aspects of surgery did not appear to account for all variation in practice recorded in our study because there was an equal amount of variation in both the explicit and tacit steps of the procedure.

There is contention within the literature as to the existence of ‘fascia’ [17] and this could explain surgeons' difficulty in articulating the origins of this tissue. The extent of variation in terminology was unexpected but is an important finding because until an agreement is made it will be difficult to conclude which technique is most effective. A previous cadaveric study has categorised the layers of the anterior vaginal wall in histological terms and identified three layers including mucosa (non-keratinised squamous epithelium overlying loose connective tissue), muscularis (smooth muscle, collagen and elastin) and adventitia [5]. When describing surgery, the use of histological terms could improve descriptions and understanding of the techniques used.

It is our interpretation that when performing a superficial dissection, this plane is between the vaginal mucosa and muscularis, a ‘deep dissection’ between the adventitia and bladder. Future research assessing the excised vaginal tissue from the anterior repair could confirm the histological level of dissection and more accurately define the 'fascia' we plicate. Video footage has shown a deeper plane to be less vascular and required minimal force, with mainly blunt dissection to develop it. This ‘deep dissection’ technique has previously been described in the literature, being the level at which mesh/grafts are placed [18]. It is likely that this technique has been extrapolated from the dissection used for insertion of graft/mesh because only PROSEPCT surgeons in this study who inserted mesh/grafts performed dissection at this depth for native tissue repairs.

The themes of surgical technique generated from this study will be used to assess the influence of surgical technique on the outcome of surgery. As well as having an understanding of how the operation varies we need to consider why surgical technique varies and this will be the subject of a further research paper.

## Conclusion

In the UK there is not a ‘standard’ native tissue anterior repair. Compared with previous questionnaire studies the use of qualitative methods has given a greater insight into the variation of surgical techniques used to perform native tissue anterior repairs. Furthermore, this study highlights the need to standardise surgical terminology. Further research is required to evaluate which anterior repair techniques are the most effective. A histological study of the excised tissue could more accurately confirm the origin of the tissue that is repaired and generically called fascia.
